# The Use of Lightweight Aggregates in Geopolymeric Mortars: The Effect of Liquid Absorption on the Physical/Mechanical Properties of the Mortar

**DOI:** 10.3390/ma17081798

**Published:** 2024-04-14

**Authors:** Emilia Vasanelli, Silvia Calò, Alessio Cascardi, Maria Antonietta Aiello

**Affiliations:** 1 CNR-ISPC (National Research Council—Institute of Heritage Science), University Campus, Prov. le Lecce Monteroni, 73100 Lecce, Italy; 2 Department of Innovation Engineering, University of Salento, University Campus, Prov. le Lecce Monteroni, 73100 Lecce, Italy; silvia.calo@unisalento.it (S.C.); antonietta.aiello@unisalento.it (M.A.A.); 3 Department of Civil Engineering, University of Calabria, Via P. Bucci, 87036 Rende, Italy; alessio.cascardi@unical.it

**Keywords:** geopolymer mortars, lightweight aggregates, sustainable mortars, thermal conductivity

## Abstract

Geopolymers have been proposed as a green alternative to Portland cement with lowered carbon footprints. In this work, a geopolymeric mortar obtained using waste materials is studied. Fly ash, a waste generated by coal combustion, is used as one of the precursors, and waste glass as lightweight aggregates (LWAs) to improve the thermal performance of the mortar. The experimental study investigates the effect of varying the alkali activating solution (AAS) amount on the workability, compressive strength, and thermal conductivity of the mortar. Indeed, AAS represents the most expensive component in geopolymer production and is the highest contributor to the environmental footprint of these materials. This research starts by observing that LWA absorbs part of the activating solution during mixing, suggesting that only a portion of the solution effectively causes the geopolymerization reactions, the remaining part wetting the aggregates. Three mixes were investigated to clarify these aspects: a reference mix with a solution content calibrated to have a plastic consistency and two others with the activating solution reduced by the amount absorbed by aggregates. In these cases, the reduced workability was solved by adding the aggregates in a saturated surface dry state in one mix and free water in the other. The experimental results evidenced that free water addiction in place of a certain amount of the solution may be an efficient way to improve thermal performance without compromising the resistance of the mortar. The maximum compressive strength reached by the mortars was about 10 MPa at 48 days, a value in line with those of repair mortars. Another finding of the experimental research is that UPV was used to follow the curing stages of materials. Indeed, the instrument was sensitive to microstructural changes in the mortars with time. The field of reference of the research is the rehabilitation of existing buildings, as the geopolymeric mortars were designed for thermal and structural retrofitting.

## 1. Introduction

Geopolymer concretes and mortars have received high attention from researchers in the last twenty years [1,2,3]. They have been proposed as a green and sustainable alternative to ordinary Portland cement with lowered carbon footprints [4,5,6]. Their use to replace Portland cement products generally results in vast energy and virgin materials savings, resulting in sustainable concrete production. Geopolymers reuse the solid waste generated in the industrial and manufacturing sectors, following the circular economy principles [7]. They are aluminosilicate-based amorphous inorganic materials obtained through a polymerization process, starting from natural or waste materials with a high content of aluminum or silicon (precursors), such as slag from blast furnaces from steel mills, clays, flying and volcanic ashes, etc. [8,9]. The reaction of the precursor powder with an alkaline activating solution (AAS), consisting of sodium and/or potassium hydroxides and silicates, at a temperature below 100 °C, produces this alkali-activated material, a robust polymer used as the binder in concretes and mortars. It has been proved that in the construction industry, the production of ordinary Portland cement is a high greenhouse gas emitter, with almost 8% of total CO_2_ production in the world [6]. Thus, the use of geopolymers represents a challenge to producing new building materials or optimizing the existing ones, reducing consumption during production, the emission of greenhouse gases, and environmental impact [10].

Besides higher sustainability, geopolymers are considered optimal candidates to substitute cement because they overcome some drawbacks. Depending on employed precursors, activating solutions, and aggregates, geopolymers may have better properties than traditional cement-based materials, like improved durability, fire resistance, and reduced thermal conductivity [11]. In particular, the literature results proved that geopolymers may be used as thermal insulating mortars and lightweight structural concrete with even better performances than cement-based materials [12,13,14]. Several methods have been reported to improve the thermal properties of geopolymers [15]: the incorporation of lightweight aggregates, the omission of fine aggregates (the no-fines concrete), and the use of foaming and air-entraining admixtures that create large bubbles and voids in concrete (the foamed or aerated concrete) [16,17]. Lightweight aggregates (LWAs) have many benefits, the most important being a high strength-to-weight ratio, but they also enhance tensile strength, and heat and sound insulation, and lower thermal expansion [15]. On the other hand, their high porosity poses some drawbacks in the mixing procedure and internal curing of the material, affecting its mechanical and physical properties. This aspect is well known from the literature on lightweight concrete mix design [18,19]. Lightweight aggregates influence more than normal weight aggregates the internal curing condition of the material, due to water absorption and interaction between lightweight aggregates and pastes. Indeed, the state of moisture of aggregates before mixing strongly influences slump loss and shrinkage of the material [20,21]. This aspect has been poorly investigated in the literature for geopolymers employing lightweight aggregates. Huiskes et al. [22] analyzed the effect of pre-soaking LWAs on the properties of geopolymer concrete. They found that pre-soaked LWAs in the mixture generated more reliable characteristics than non-pre-soaking mixtures in terms of stability, compaction, air content, porosity, and particle distribution. Kupaei et al. [23] added oil palm shells (OPS) as lightweight aggregates in two moisture conditions: the air dry (AD) and saturated surface dry (SSD) condition. They found that the compressive strength development was dependent on the conditions of OPS. The mixes with air-dry OPS produced a lower strength of about 7% compared to the mixes with OPS in the SSD condition.

The present study wants to deepen the effects of liquid absorption by LWA on the final properties of a geopolymeric mortar. It is part of extensive research aiming at developing a geopolymeric mortar for the seismic/thermal retrofitting of existing buildings, from the perspective of the circular economy. The mortar employs waste materials as aggregates and precursors. The precursor is fly ash, a waste generated by coal combustion and widely used as an additive in the building sector. The aggregates were expanded waste glass particles of different grades. They were used as LWAs to improve the thermal insulating properties of the geopolymeric mortar. The LWAs usually absorb water when employed in dry/ambient conditions in concrete mixes. In the case of a geopolymer, the liquid part of the mix is the alkaline activating solution, which is indispensable to start the geopolymerization reactions. When LWAs are added to the mix at ambient conditions, part of the AAS is absorbed by aggregates [22,23]. Thus, it can be supposed that only part of the solution effectively causes the geopolymerization reactions to take place as the remaining part wets the aggregates and ensures the necessary workability of the mix. This circumstance is antieconomic if it is considered that the alkaline solution is expensive and has a high impact on the total cost of the material. Furthermore, it has been proved that AAS has a higher environmental impact regarding categories other than global warming, namely freshwater, terrestrial, and marine aquatic ecotoxicity potential, ozone depletion potential, and human toxicity potential [4,6,24]. Based on these considerations, this paper wants to investigate the effects of reducing the content of the AAS by subtracting the amount that aggregates absorbed during mixing. To this scope, three formulations were made: a reference mix in which the content of the AAS was sufficient to obtain a plastic consistency of the mortar and two other mixes with a reduced content of the activating solution. In these cases, the subsequent reduced workability was solved in two ways: by adding free water (FW mix) or aggregates in the SSD state (SSD mix). The workability, compressive strength, and thermal conductivity were measured to compare the properties of the SSD and FW mixes with the reference one.

## 2. Materials and Methods

### 2.1. Materials

Three geopolymeric mortar formulations were developed. All the mixes were obtained by mixing the precursors (fly ash and metakaolin) with the alkali-activating solution and then adding LWAs.

The composition obtained by SEM-EDS analysis and the loss of ignition (LOI) of fly ash and metakaolin are reported in Table 1.

The alkali-activating solution was made of sodium silicate (SS) solution (Extra Pure by Merck KGaA, Darmstadt, Germany) and sodium hydroxide solution (SH). The last was obtained by dissolving sodium hydroxide in distilled water to have a twelve-molar solution (12 M).

Lightweight aggregates employed in the study were commercially available (by Poraver, Schlüsselfeld, Germany) and made of expanded waste glass particles. Five different grain sizes were used (0.04–0.125 mm, 0.1–0.3 mm, 0.25–0.5 mm, 0.5–1 mm, 1–2 mm) and graded according to the Fuller’s distribution. The LWA dry loose bulk densities vary between 230 kg/m^3^ (for the coarsest fraction) and 530 kg/m^3^ (for the finest fraction).

The geopolymer mortars were formulated maintaining the following fixed proportions among the ingredients, based on previous research by the authors and the literature findings [25,26].

- The weight of metakaolin was 10% of the total weight of precursors.
- The weight ratio SS/SH was 2.5.
- The aggregate-to-binder volume ratio was 0.95 corresponding to 40% in weight.

The three geopolymer mixes differed in the alkali-activating solution and water contents. The reference mix is called SOL because the liquid part of the mix was only the activating solution without water addiction. In the SOL mix, the AAS-to-binder ratio was 0.67. This value was obtained after preliminary tests to guarantee the plastic consistency of the mortar.

The other two mixes were developed to reduce the amount of the alkaline solution by the quantity absorbed by aggregates during mixing. It was estimated starting from the technical datasheet of aggregates. The supplier furnished the water absorption of each aggregate fraction to reach a saturated surface dry condition (SSD). Considering the amounts of each fraction given by Fuller’s distribution, a water absorption of about 20% of the total weight of aggregates was calculated. The corresponding amount of activating solution was estimated to be 30% of the LWA weight, accounting for the difference in density between water and activating solution (1000 kg/m^3^ and 1325 kg/m^3^, respectively). Based on this value, a solution-to-binder ratio equal to 0.55 was obtained. Thus, the other two mixes (SSD and FW) were designed considering this ratio. In the SSD mix, the aggregates were added in SSD conditions; in the FW mix the aggregates were at ambient conditions, and free water was added. The water content was equal to the quantity necessary to bring aggregates in the saturated surface dry condition according to the values reported in the datasheet. The resulting mix proportions are reported in Table 2.

### 2.2. Mixing and Curing 

The AAS was prepared 24 h before mixing. The solution was added gradually to dry precursors in a Hobart mixer at 140 rpm. After the solution addiction, the mixer continued to work for another five minutes at the same speed. The lightweight aggregates were then added. They were at ambient conditions for the SOL and FW mix, while for the SSD mix, aggregates were preliminarily conditioned to have a saturated surface dry condition. They were immersed in tap water for 24 h before mixing and drained on a sieve to eliminate water. Then, they were dabbed on the surface with filter paper to reach the SSD condition. The cone procedure described in [27] was carried out to verify the SSD state. Free water was added to the FW mix after aggregate addiction. After adding aggregates and free water to the FW mix, the mixer was run for another five minutes. The mortars were manually mixed with a spatula before being placed into molds to ensure the homogeneity of the mixture. Specimens were de-molded after two to five days, depending on their consistency, and then cured at laboratory conditions (23 °C, 50% RH).

### 2.3. Test Methods

The consistency of the mortars was measured immediately after mixing by a flow-table test [28]. The mold was filled in two layers 25 mm thick. Each layer was consolidated with 25 times tamping of the flow table before measurements. The mold was removed to let the mortar flow. Two perpendicular diameters were measured to determine the mean diameter of the sample (flowability) expressed in millimeters.

Apparent density was monitored during curing up to 48 days from casting. It was determined on four specimens 40 × 40 × 80 mm for each mix by dividing their mass by the volume. The last was calculated by measuring each side of the specimens at four different points using a caliper. All the measurements were carried out under laboratory conditions and not after oven conditioning to avoid the influence of temperature on the kinetic of geopolymerization reactions and the water vapor evaporation from specimens.

Ultrasonic pulse velocity (UPV) measurements were carried out in direct transmission mode using 150 kHz transducers (Pundit PL 200, Screening Eagle Technologies AG, Schwerzenbach, Zurich). UPVs were measured at the timescale and on the specimens used for density measurements. Four measurements were taken, and the average value was calculated for each specimen (along the 40 × 80 faces).

After 48 days from casting, all the specimens were broken under compression test to determine the compressive strength of the materials [29]. The compressive strength was calculated as the mean value of the four measurements at a loading rate of 200 N/s. 

The thermal conductivity of the mortars was measured on disk-like specimens, 50 mm in diameter (FOX 50 Laser Comp—TA Instruments, New Castle, DE, USA) according to [30,31]. The two-thickness method was used. It had the best precision guaranteed by the instrument producer (3%) and allowed for estimating the specimen’s thermal conductivity and contact thermal resistance. The two thicknesses were 15 mm and 6 mm. Specimen surfaces were lapped to minimize the surface roughness and ensure good contact with the instrument plates. The upper plate temperature was set to 15 °C and the lower plate to 25 °C. Two couples of specimens were tested for each batch, and the average value was calculated. The specimens were in ambient conditions when tested.

## 3. Results and Discussion

### 3.1. Consistency of the Mortars

It is known from the literature that the content of water and activating solution strongly affects the workability of the geopolymers: generally, the higher the liquid-to-binder ratio, the higher the workability of the mix [32,33,34,35]. The results of the present study followed the results of the literature, as the consistencies of the three mixes were very different depending on the content of the activating solution and water.

The consistency of the SOL mix was plastic, with a flow value of 180 mm. The value is comparable to those of commercially available mortars for composite-reinforced mortar application, and it is suitable for application on building vertical surfaces. The SSD mix had a higher flowability (>300 mm) than the SOL mix. The result can be explained by the saturated surface dry condition of aggregates, which caused the activating solution to be not absorbed by aggregates during mixing and be available to provide workability [22]. To effectively compare the liquid-to-binder ratio of SOL and SSD mixes, the aggregates should be considered in the same conditions. Thus, the liquid content of the SSD mix should be calculated as the sum of the activating solution and water absorbed by aggregates after dabbing. In this case, the liquid-to-binder ratio of the SSD mix would be 0.83 in contrast with 0.67 of the SOL mix. The values explain the higher flowability of the mix. The amount of water absorbed by aggregates with the adopted SSD procedure was much higher than that reported in the datasheet of aggregates, probably due to the difficulties in effectively drying the surface of the finest aggregate fraction [36]. More research is needed to clarify this point and optimize the SSD procedure for the finest fractions of the mix. 

As regards the FW mix, a 245 mm flow was registered. This value was higher than that of the SOL mix, even if the total liquid content (SS + SH + W) and the ratio of liquid to binder ((SS + SH + W)/(FA + MK)) were slightly lower (Table 2). The difference in viscosity between water and the activating solution may explain the result. It is reported in the literature that at the same liquid content, the ratio of SS solution on SH strongly influences workability [32,35,37]. It is explained by the higher viscosity of the SS solution compared to that of SH and water, which reduced the workability [36,38]. The SOL and FW mixes had the same SS-to-SH ratio, but the content of SS in the SOL mix was sensibly higher than in the FW mix, as it was 72% of the total liquid content instead of 62% for the FW mix.

### 3.2. Density and UPV Monitoring

**Figure 1:**
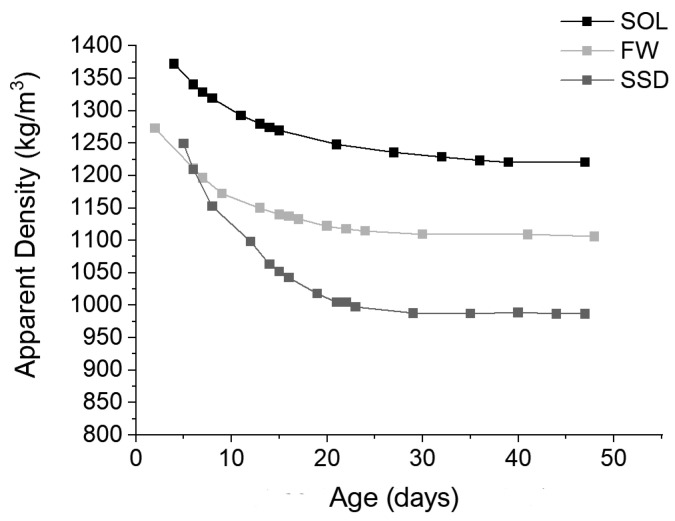
Apparent density vs age of the mortars (days), measured in SOL, FW, and SSD mixes.

The apparent density of the geopolymers decreases with curing time, especially during the first ten days (Figure 1). Indeed, water contained in the activating solution, free water, or water produced during the geopolymerization reactions evaporates with time [33,39,40] in all the mixes, causing a density reduction. At the age of 32 days, the density of the mixes became constant. Before this time, it decreased with different speeds depending on the mix.

SOL and FW behaviors with time were similar. Up to ten days, the rate of density decrease was higher, and then it diminished in both the mixes to become constant after 30–32 days from casting. The SOL density was higher than the FW density because of its higher content of activating solution, following the results of consistency tests. 

The SSD behavior was different in the first days of curing. Because of its very fluid state, it was possible to demold specimens only after five days from casting. Up to 20 days, the mix lost about 20% of the initial density, contrasting with 12% and 10% of FW and SOL mixes, respectively. The SSD mix had a lower density than the other two mixes, following the difference in consistency among mixes.

The residual water inside the specimens was estimated using the following equation [39]:

(1) $$Residual\;water\;(\%)=\frac{W_{H_{2}O}-{W^{\prime }}_{H_{2}O}}{W_{n}}$$

where *W*_*H*_2_*O*_ was the initial weight of the water in the specimen, *W*′_*H*_2_*O*_ was the total water loss by the specimen on the day of measurement starting from the day of demolding, and *W_n_* was the weight of the specimen on the day of measurement. The initial weight of water was determined as the sum of the content of water contained in SS and SH, the free water in the case of the FW mix, and the water absorbed by aggregates in the case of the SSD mix.

**Figure 2:**
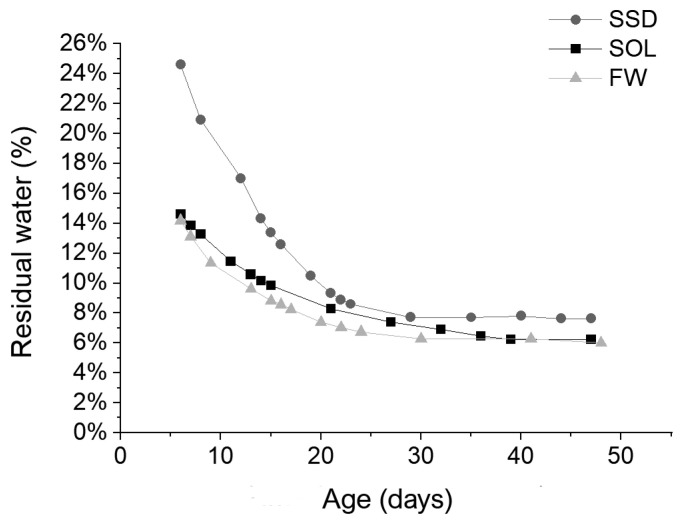
Residual water (%) vs mortar age (days) calculated for SOL, FW, and SSD mixes.

The results (Figure 2) were interesting as they showed that different mixes reached a similar value of the residual water in the material independently of the initial amount of water and solution in the mix. 

**Figure 3:**
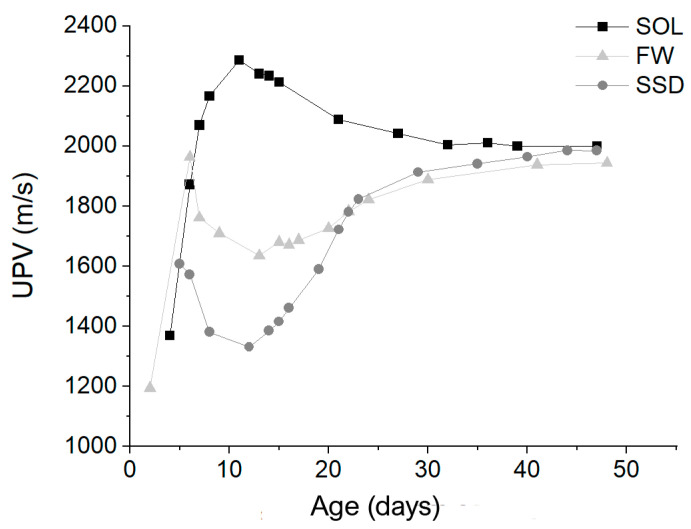
UPV values vs mortar age (days) calculated for SOL, FW, and SSD mixes.

The ultrasonic pulse velocity measurements were carried out frequently during the curing period, especially in the first days after demolding (Figure 3). The measurements highlight some topics that, to the author’s knowledge, were not evidenced in the literature. The literature on the UPV application on geopolymers shows that UPV increases with time due to the progression of geopolymerization reactions [41,42]. The measurements were generally performed on fixed dates, like 7, 14, and 28 days from casting. In the present study, measurements were performed on several dates after demolding, evidencing different stages in UPV variation with time. Indeed, in all the mixes UPV first increased and then decreased. UPV increased again in the FW and SSD mixes while they remained constant in the SOL mix. In the last stage of measurements, UPV also remained constant for the FW and SSD mix.

The UPV behavior with time may be explained by considering two contemporaneous effects in the geopolymers curing. 1. The decrease in density with time due to water evaporation; 2. the hardening of the material with time due to the progression of the geopolymerization reactions. Water evaporation caused a substitution of water with air within the material pores, determining a UPV reduction. In contrast, the hardening of the material with the geopolymerization reaction caused UPV to rise. These two effects and the prevalence of one over the other determined the trend of UPV measurements.

Four stages can be distinguished (Table 3, Figure 3).

- Stage 1: in the first days from casting, the increase in stiffness prevailed, even if density decreased faster in this period (Figure 1). In fact, during this stage, a UPV increase can be observed, characterized by a longer duration for SOL (11 days) than for FW and SSD (6 and 5 days, respectively).
- Stage 2: the effect of density reduction prevailed, causing a UPV lowering. During this stage, the geopolymerization reactions slowed down, accompanied by prevailing water evaporation. This stage lasted longer for the SOL mix (about 20 days) than for FW and SSD mixes (about one week). The rate of UPV reduction of the SOL mix slowed down after the 21st day, following the density behavior (see Figure 1). The FW and SSD mixes behave differently. The duration of this stage was shorter and followed by stage three, not experienced by the SOL mix.
- Stage 3: This stage was visible only for SSD and FW mixes. The UPV increased in both of the mixes due to the material hardening. The increment had two phases with different rates, first higher and then lower (Table 3). For the SSD mix, the UPV increase after the 12th day was considerable: 50%, in contrast with 20% for the FW mix.
- Stage 4: UPVs remained constant in all the mixes. This stage started earlier for the SOL mix (32nd day) and later in FW (41st day) and SSD (44th day) mixes.

Considering the UPV trends in the mixes, it can be deduced that most of the hardening of the SOL mix was until the 11th day from casting. In the other two mixes, hardening of the materials happened in the first week, then it stopped for some days and started again after about 12–13 days from casting. The higher ratio of activating solution to binder ratio of the SOL mix may have contributed to the faster development of the geopolymerization reactions [2,35,36]. It is reported in the literature that an excess of water may cause a delay in the progression of the geopolymerization reactions [36,40,43]. It probably happened in the FW and especially in the SSD mix following the higher flowability of the two mixes compared to the SOL mix. 

At the end of UPV monitoring, the three mixes had similar UPV values.

### 3.3. Compressive Strength and Thermal Conductivity

It is well known from the literature that the compressive strength of geopolymers depends on several factors: type of precursors, curing conditions, molarity of NaOH solution, SS/SH ratio, alkaline solution-to-binder ratio, the content of free water, and aggregate-to-binder content are the most cited [22,37,40,44,45]. Most of these factors, namely the aggregates’ content, the NaOH solution’s molarity, and the SS/SH ratio, were constant in this study. In contrast, the alkaline solution content was varied among the mixes. 

**Figure 4:**
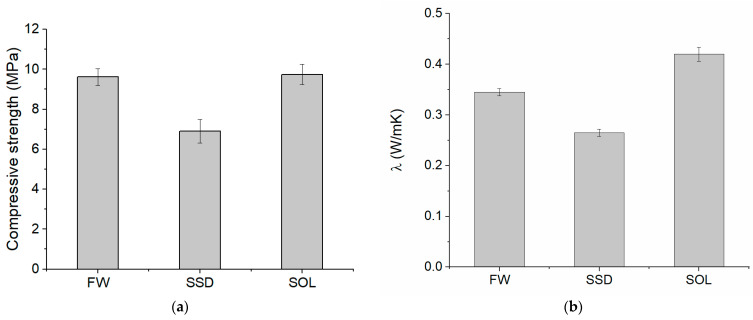
Compressive strength (**a**) and thermal conductivity λ (**b**) of the investigated mortars.

The compressive strength of the mortars was measured at the age of 48 days when density and UPV were constant for all the mortars. Testing mortars at this date and not at 28 days, as usual, ensured a proper comparison of the mechanical properties of different mixes because most of the geopolymerization reactions occurred in all of them. Indeed, after 28 days, while the SOL mix had almost stable values of UPV, both the FW and SSD mixes still had UPV rising with time. The compressive strengths of the FW and SOL mixes were comparable (Figure 4a), even if the content of the alkaline solution was different. The alkaline solution-to-binder ratio was 0.67 and 0.55, respectively, for the SOL and the FW mixes. Thus, the compressive strength results showed that a ratio of 0.55 was sufficient to cause geopolymerization reactions and the consequent material stiffening. Replacing part of the solution with water in the FW mix guaranteed a higher flowability than the SOL mix without compromising the compressive strength. Anyway, the threshold of water content in substitution of the activating solution that does not compromise strength should be assessed by further investigations. Indeed, it is reported in the literature that increasing water instead of the solution may bring adverse effects in resistance [35,37].

The SSD mix had a lower compressive strength than the other two mixes. This result cannot be attributed to the content of the activating solution, as it was the same as that of the FW mix. The lower compressive strength result followed the higher flowability and lower density results, and it is due to the different behavior of aggregates in the SSD state. As stated before, the total liquid-to-binder ratio of the SSD mix was higher than those of the other two mixes, causing a higher flowability, lower density, and lower compressive strength of the mix. Thus, the optimization of AAS content or the procedure to obtain LWAs in SSD conditions should be performed to reduce the flowability of the SSD mix and eventually reach the resistance of the other two mixes. More research is needed to deepen this aspect.

Thermal conductivity as compressive strength was affected by the content of the activating solution as the results varied with mixes (Figure 4b). SSD had the lowest thermal conductivity (0.27 W/mK), and the SOL mix the highest (0.42 W/mK). The thermal conductivity of the FW mix was between the values of the other two mixes (0.34 W/mK).

**Figure 5:**
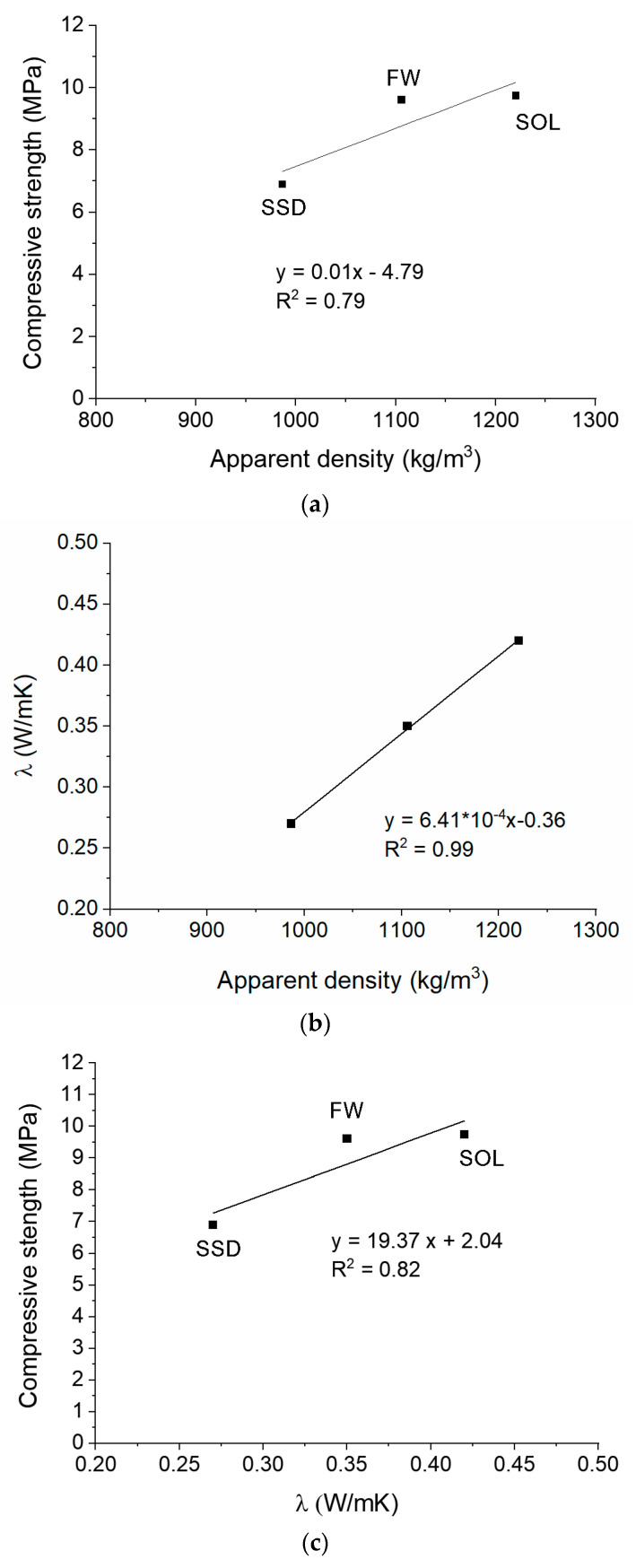
Linear correlations between compressive strength and density (**a**), thermal conductivity and density (**b**), and compressive strength and thermal conductivity (**c**).

Both thermal conductivity and compressive strength strongly depend on the density of the mixes, following the literature findings [15,22,32,46]. In Figure 5, linear correlations between the density (Figure 1), compressive strength (Figure 4a), and thermal conductivity (Figure 4b) results are reported (Figure 5). The high coefficient of determination of the obtained correlations evidenced a strong dependence among results. The best linear fit was between the thermal conductivity and the density results. All these properties depend on the porosity of the mixes, which in turn depends on the content of the liquid part of the mix and consistency. Thus, the higher flowability of the mix caused a higher porosity and, consequently, lower density, compressive strength, and thermal conductivity. The obtained correlations are not exhaustive due to the low number of data but are indicative of the dependence among the investigated properties of the mortars.

## 4. Conclusions

This work is part of extensive experimental research that aims to optimize a geopolymer mortar with good mechanical and thermal performances. The mortar is formulated to be a sustainable material, as it employs waste aggregates and precursors, and it does not contain cement, which is considered the largest gas emitter in the construction industry. Fly ash, a waste of the coal industry, was employed as a precursor and waste glass as lightweight aggregates to improve the thermal performance of the mortar. 

This experimental study investigates the effect of varying the content of the activating solution on the workability, compressive strength, and thermal conductivity of the material. This work starts from the observation that the expanded glass aggregates absorbed a consistent part of the activating solution during mixing. Thus, it can be supposed that only part of the solution effectively causes the geopolymerization reactions while the remaining part is needed to wet the aggregates, guaranteeing workability. This circumstance is antieconomic if it is considered that the alkaline solution is expensive and increases the environmental impact of the material. 

Three mixes were investigated to clarify these aspects. The SOL mix was the reference mix in which the AAS content was calibrated to have a plastic consistency of the mortar. The other two mixes (SSD and FW mixes) had their AAS lowered by the quantity absorbed by aggregates. In these cases, two strategies were considered to overcome the reduced workability: in the SSD mix, aggregates were added in a saturated surface dry state; in the FW mix, the aggregates were at ambient conditions, and free water was added. 

The three mixes had different properties due to the differences in the contents of water and AAS. The SOL mix had the highest density, compressive strength, and thermal conductivity. The FW mix had a compressive strength comparable to the SOL mix, a lower density, and a lower thermal conductivity. The SSD mix had the lowest compressive strength, density, and thermal conductivity.

The comparable mechanical results of FW and SOL mixes mean that replacing part of the activating solution with free water may be a way to obtain better thermal performances without compromising the resistance of the mortar. On the other hand, more research is needed to find the optimal water content to compromise the mechanical performance and thermal efficiency of the material. 

Regarding the SSD mix results, the mix cannot be considered optimized as its consistency was too fluid. It was probably due to difficulties obtaining the finest fractions of LWA in SSD states as they absorbed too much water. Optimizing the procedure or reducing the AAS content may allow for better performances of the SSD mix in flowability and mechanical resistance. More research is needed to investigate this aspect.

Another finding of the experimental research is using UPV to follow the curing stages of materials. Indeed, the instrument was sensitive to microstructural changes with time. The result is noteworthy because UPV reduces the number of samples that would otherwise be required for destructive testing to monitor the hardening progress of the material over time.

The obtained experimental results concern a limited number of samples, so this research is not intended to be exhaustive of the topics covered; it is considered exploratory for the subsequent experimental investigations. Indeed, the experimental results should be further improved by increasing the number of tested specimens and deepening other aspects that have not been faced, like the influence of the investigated parameters on the shrinkage, porosity, and durability of the different mixes. These aspects will be the object of future research. 

## Figures and Tables

**Table 1:** Fly ash and metakaolin compositions obtained by SEM-EDS.

	Oxides Concentration %									LOI %
	SiO_2_	CaO	Al_2_O_3_	Fe_2_O_3_	K_2_O	TiO_2_	SO_3_	Na_2_O	MgO	
Fly ash	59.54	1.76	27.26	2.91	2.71	0.59	1.02	2.56	1.65	3.20
Metakaolin	56.50	2.51	27.92	0.77	1.83	0.44	0.29	9.03	0.71	1.29

**Table 2:** Mix proportioning of the geopolymeric mortars. FA: fly ash; MK: metakaolin; SS: sodium silicate solution, SH: sodium hydroxide solution; LWA: lightweight aggregate; W: water in the mix, added as free water in FW mix and absorbed by aggregates in SSD mix.

Mix Nomenclature	FA + MK (kg/m^3^)	SS + SH (kg/m^3^)	LWA (kg/m^3^)	W (kg/m^3^)
SOL	504.84	337.35	202.73 *	-
SSD	529.13	289.84	361.22 **	148.73
FW	507.56	278.02	203.82 *	40.76

* Ambient conditions; ** SSD condition.

**Table 3:** Four stages of UPV behavior: periods of duration. In brackets are the dates in which there was a change in the rates of UPV increase/decrease.

Mix	Stage 1	Stage 2	Stage 3	Stage 4
FW	Up to 6th day	6th–(7th)–13th day	13th–(30th)–41st day	41st day
SSD	Up to 5th day	5th–(8th)–12th day	12th–(23rd)–44th day	44th day
SOL	Up to 11th day	11th–(21st)–32nd day		32nd day

## Data Availability

Data are contained within the article.
